# The Effect of Lemon Inhalation Aromatherapy on Nausea and Vomiting of Pregnancy: A Double-Blinded, Randomized, Controlled Clinical Trial

**DOI:** 10.5812/ircmj.14360

**Published:** 2014-03-05

**Authors:** Parisa Yavari kia, Farzaneh Safajou, Mahnaz Shahnazi, Hossein Nazemiyeh

**Affiliations:** 1 Department of Midwifery, Faculty of Nursing and Midwifery, Tabriz University of Medical Sciences, Tabriz, IR Iran; 2 Nanotechnology Research Center, Faculty of Pharmaceutical, Tabriz University of Medical Sciences, Tabriz, IR Iran

**Keywords:** Nausea, Vomiting, Aromatherapy, Citrus

## Abstract

**Background:**

Nausea and vomiting of pregnancy are amongst the most common complaints that effects on both the physical and mental conditions of the pregnant women. Due to the increasing tendency of women to use herbal medications during pregnancy, the effect of lemon inhalation aromatherapy on nausea and vomiting of pregnancy was investigated in this study.

**Objectives:**

The aim of this study was to determine the effect of lemon inhalation aromatherapy on nausea and vomiting during pregnancy.

**Materials and Methods:**

This was a randomized clinical trial in which 100 pregnant women with nausea and vomiting who had eligibility criteria were randomly divided into intervention and control groups based on four- and six-random block sampling method. Lemon essential oil and placebo were given to the intervention and control groups, respectively, to inhale it as soon as they felt nausea. The nausea, vomiting, and retch intensity were investigated 24 hours before and during the four days of treatment by means of PUQE-24 (24-hour Pregnancy Unique Quantification of Emesis).

**Results:**

There was a statistically significant difference between the two groups in the mean scores of nausea and vomiting on the second and fourth days (P = 0.017 and P = 0.039, respectively). The means of nausea and vomiting intensity in the second and fourth days in the intervention group were significantly lower than the control group. In addition, in intragroup comparison with ANOVA with repeated measures, the nausea and vomiting mean in the five intervals, showed a statistically significant difference in each group (P < 0.001 and P = 0.049, respectively).

**Conclusions:**

Lemon scent can be effective in reducing nausea and vomiting of pregnancy.

## 1. Background

Nausea and vomiting of pregnancy (NVP) are amongst the most common complaints of women during pregnancy that 50 to 80 percent of women have experienced various degrees of it (1). The beginning of NVP is highly variable and is usually between the first and second missed menstrual cycle; it continues to 14 to 16 weeks of pregnancy (2) and usually has the most severity form 7 to 9 weeks of pregnancy (3). In 50% of women, NVP resolves by the 14 weeks and in 90 percent of women by the 22 weeks (2).

NVP not only has adverse effects on women's physical health but also has negative effects on psycho-social performance of them (1, 4). Loss of work days, lack of energy, fatigue, irritability, lack of enjoyment of life, and lack of preparation for childbirth can cause considerable stress on women (5). Since the pathophysiology of NVP is not well understood, many available treatments are just prescribe to reduce symptoms. NVP treatment depends on the severity of symptoms and varies from changes in diet and lifestyle to hospitalization (5). The results of a review study in 2010 by the Cochrane database showed that there were limited evidence supporting the use of medications such as vitamin B6 and antiemetic medications to relieve mild or moderate NVP. In addition, for non-pharmacological methods, the evidences on the effectiveness of acupressure were limited and the use of acupuncture in pregnant women showed no significant benefits. The use of ginger products may also be useful but there are limited evidences on the effectiveness of it (6).

Nowadays, the tendency in women to use non-medicinal and herbal products in pregnancy has increased due to concerns about drugs adverse effects in early pregnancy (6). According to a study, 49.2% of women during pregnancy used herbal medicines; 39.3% of them had used these medication to gastrointestinal problems of which 5.71% were due to NVP (7). The majority of midwives in Iran use aromatherapy, phytotherapy, and massage more than any other non-pharmacological methods that is due to the popularity of these methods and their practical experience in such methods (8). Among the non-pharmacological approaches, aromatherapy can be noted. Aromatherapy, which is a branch of herbal science, is the collection of methods for skillful and controlled use of essential oils to promote the physical, emotional, and psychological health (9).

Lemon essential oil (Citrus lemon) is one of the most widely used herbal oils in pregnancy and is considered as a safe drug in pregnancy. One or two drops of lemon essential oil in an oil burner or a diffuser in bedroom helps to soothe and relieve NVP (10). According to a study, 40% of women have used lemon scent to relieve nausea and vomiting, and 26.5% of them have been reported it as an effective way to control their symptoms (11). Smith et al. also have been considered the fresh lemon smell helpful for NVP (12). Due to the increasing interest in the use of herbal drugs in pregnancy, availability of lemon in all seasons, and its high range of use in Iranian society, this study aimed to determine the effect of lemon inhalation aromatherapy on NVP in the health centers of Birjand, Iran.

## 2. Objectives

The aim of this study was to determine the effect of lemon inhalation aromatherapy on NVP.

## 3. Materials and Methods

This study is a randomized clinical trial carried on 100 pregnant women with NVP who were referred to the health-medical centers of Birjand city, Iran. These pregnant women had mild to moderate nausea, with or without vomiting and six to 16 weeks gestation, singleton pregnancy, without signs of threatened abortion and any other disease with nausea and vomiting as a symptom, and without any antiemetic drug use in the past 24 hours. To determine the sample size, we used the formula of comparison of two means. Due to lack of access to study that M1 and SD1 can be extracted from, initially a pilot study was conducted on 30 patients. Using the numbers in this study (M1 = 7.36, SD1 = 1.82), by taking the mean difference at least 20%, α = 0.05, power 90%, and included 10% loss, the sample size of 50 in each group was calculated. After obtaining permission from the Research Ethic Committee of Tabriz University of Medical Sciences (code: 916) and after explaining the purpose and methodology of the study, eligible patients who tended to participate entered the assessment phase for enrolment in the study. The participants were asked to answer the PUQE-24 questionnaire, related to the assessment of NVP in past 24 hours, and if their scores were between 3-12 (mild to moderate nausea and vomiting), they were enrolled in the study. After taking written informed consent, the participants were asked to fill out questionnaires about their demographic and pregnancy characteristics.

PUQE-24 questionnaire, which is designed to measure the severity of NVP, is composed of three questions that measures nausea duration and frequency of vomiting and retch in the last 24 hours through a five-point Likert scale. The range of scores for each question is from one to five points and for the total score is between 3 and 15; the score ≤ 6 indicates mild nausea, 7-12 moderate nausea and vomiting, and ≥ 13 indicates the severe type (13). To determine the validity of the questionnaire, content validity method was used; after the translation of the questionnaire, they were delivered to 10 members of the Faculty of Medical Sciences of Tabriz University, and after gathering opinions and revisions, it was used for the study. The Cronbach's alpha coefficient was used to assess the reliability of the method; Cronbach's alpha was 0.81. Allocating the participants in the experimental group (the group that inhaling lemon) or control group (group that would inhale placebo) was done in the random allocation method using computerized random number table and the four and six blocking method with allocation ratio of 1:1.

For allocation concealment, dark and similar packaged containers sequentially numbered from one to 100 were used. It was executed by a person uninvolved in the study. The containers in the intervention group contained 10 cc of lemon oil, produced in Tabriz Pharmaceutical Nanotechnology Research Center. The essential oil was prepared form the lemon peel and in solvent distillation method and almond oil was used as a carrier oil. To make placebo, the colors of carrots (for being in the same color with lemon oil) was used in combination with almond oil. The researcher and participants were unaware of the content of the containers.

The participants in both study groups must had the advantage of receiving nutritional recommendations as well as recommendations relating to lifestyle changes which were proven to control nausea and vomiting; hence, recommendations were presented both in printed form and orally to all participants. Two groups of participants were asked to follow the nutritional recommendations and lifestyle changes, and when they felt nauseated, they had to drop two drops (10) of the solution on the cotton, and keep it in distance of 3cm of their nose, and then breathe three times deeply through the nose (14). If necessary, they had to repeat it five minutes later (15). It should be noted that the cottons given to the participants were in the same size. 

Four copies of PUQE-24 questionnaire were given to the participants to fill in each day of study. A telephone number was given to every participant for help them during treatment, and the participant were followed by phone about the ways of filling out the questionnaires or the treatment and their questions were answered. After four days, the questionnaires were collected and the final questionnaire was filled through interviews with participants. This questionnaire contained information on treatment satisfaction of participants, the presence or absence of complications, and the degree of compliance with the recommendations related to the changes in the diet and lifestyle.

Normality of the quantitative variables in each group was reviewed and upheld through descriptive tests. To compare qualitative variables in the two groups the Chi-square (χ2) test with accurate P-value, and in case of ranking of the variables, the Chi-square trend was used. To compare the nausea and vomiting variables between the two groups before the intervention the t-test was used, and to compare the mean scores for nausea and vomiting between the two groups after the intervention, the ANCOVA statistical test, with adjusting the baseline and confounding variable (gravidity) were used. In order to measure the variation at the time of measurements in each group for the severity of nausea and vomiting, the analysis of variance with repeated measures was used. The data analysis was performed using software SPSS v.13, and P value < 0.05 was considered as statistically significant.

Clinical trial registration code: 201202297418 N2 

## 4. Results

The study included 100 participants in two groups of 50 women. Data collection lasted ten months (8/2012-5/2013). During the study, there was no loss of sample due to drop out, missed to follow-up or voluntary exit from the study and all the participants continued their cooperation to the end of the research. Demographic and pregnancy characteristics are presented in Tables 1 and 2. There were no significant differences between two groups in terms of demographic and pregnancy, except for gravidity. First pregnancies composed 68% and 42% of pregnancies in the intervention and the control group, respectively, which showed statically significant difference between the two groups (P = 0.028); hence, this variable was adjusted as a confounding factor. The results showed that by controlling nausea and vomiting before intervention and confounding variables (gravidity), there was statistically significant differences between the mean scores of nausea and vomiting on the second and fourth days in two groups. The mean nausea and vomiting scores on the second and fourth days in the intervention group were significantly lower than in the control group; however, the difference was not significant on the first and third days (Table 3). 

**Figure 1.**
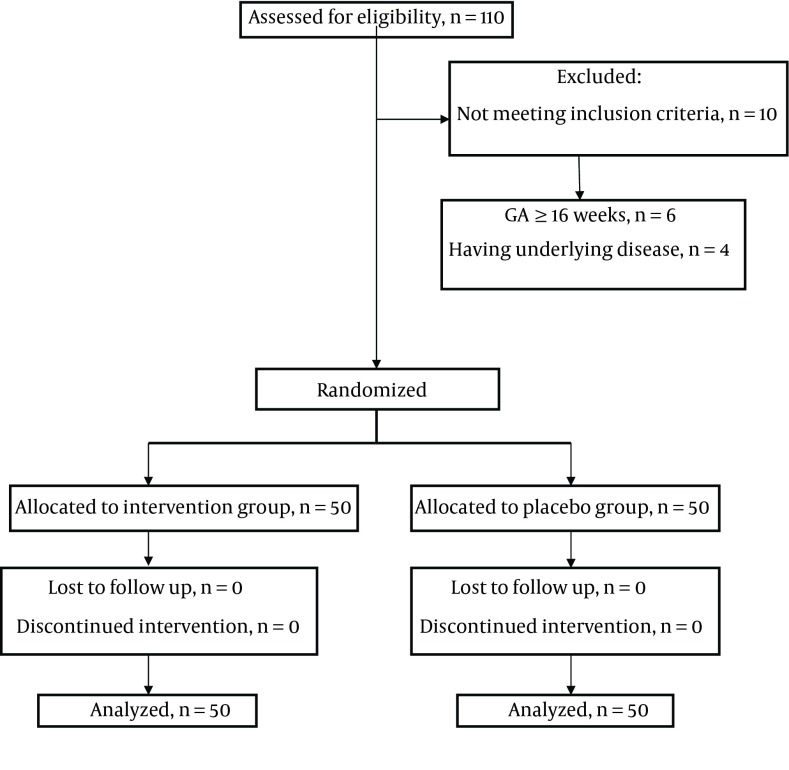
Flow Diagram of Participants Through Each Stage of Randomized Controlled Trial

Analyses of the data by ANOVA with repeated measures showed that the decrease in the mean scores for nausea and vomiting in five assessed intervals was statistically significant in both groups. This reduction in the intervention group was more considerable than the control group. Mean difference score for nausea and vomiting before and four days after the intervention in two groups was statistically significant (Table 4). In the intervention group, 50% of participants were satisfied with the given treatment, while this ratio in the control group was 34% and in this respect, there was significant difference between the two groups (P = 0.015). In terms of frequency of drug use, the majority of the intervention group (56%), had used the drug four to six times a day, and the majority of the control group (52%), one to three times a day, which showed no significant difference between the study groups (P = 0.277). All participants in the study had adhered to recommendations for changes in the diet and lifestyle and in both groups. There was no adverse effects due to treatment (Figure 1). 

**Table 1.** Demographic Characteristics of the Two Groups^ a^

	Intervention Group (n = 50)	Control Group (n = 50)	Total (n = 100)	Statistical Indicators
**Age, y**	26.2 ± 5.58	25.76 ± 4.65		T ^b^ = 0.44 , df = 98, P = 0.65
**Education**				χ2 ^c^ = 0.85, df = 1, P = 0.35
Elementary	13 (26)	16 (32)	29 (29)	
Middle school	11 (22)	10 (20)	21 (21)	
High school and Diploma	15 (30)	18 (36)	33 (33)	
University	11 (22)	6 (12)	17 (17)	
Total	50 (100)	50 (100)	100 (100)	
**Job Status **				χ2 = 3.51, df = 2, P = 0.17
House keeper	45 (90)	44 (88)	89 (89)	
Employed outside home	5 (1)	3 (6)	8 (8)	
Working at home	0 (0)	3 (6)	3 (3)	
Total	50 (100)	50 (100)	100 (100)	
**Income**				χ2 = 1.34, df = 2, P = 0.51
Earn equal pay	35 (70)	34 (68)	69 (69)	
Earn less pay	12 (24)	15 (30)	27 (27)	
Earn more pay	3 (6)	1 (2)	4 (4)	
Total	50 (100)	50 (100)	100 (100)	
**Smoking husband**				Fisher Exact Test = 0.00, df = 1, P = 1
Yes	3 (6)	3 (6)	6 (6)	
No	47 (94)	47 (94)	94 (94)	
Total	50 (100)	50 (100)	100 (100)	

^a^ Data are presented as mean ± SD or No. (%)

^b^ Independent sample t-test

^c^ Trend χ2

**Table 2.** Pregnancy Characteristics of the Two Groups ^a^

	Intervention Group (n = 50)	Control Group (n = 50)	Total (n = 100)	Statistical Indicators
**Gravidity**				χ2 = 4.82, df = 1, P = 0.028
1	34 (68)	21 (42)	55 (55)	
2	9 (18)	18 (36)	27 (27)	
≥ 3	7 (14)	11 (22)	18 (18)	
Total	50 (100)	50 (100)	100 (100)	
**Wanted pregnancy**				Fisher Exact Test = 1.51, df = 1, P = 0.35
Yes	46 (92)	42 (84)	88 (88)	
No	4 (8)	8 (16)	12 (12)	
Total	50 (100)	50 (100)	100 (100)	
**Importance of gender of baby**				Fisher Exact Test = 2.10, df = 1, P = 0.21
Yes	15 (30)	22 (44)	37 (37)	
No	35 (70)	28 (56)	63 (63)	
Total	50 (100)	50 (100)	100 (100)	
**Medication before intervention**				Fisher Exact Test = 1.50, df = 1, P = 0.326
Yes	13 (26)	8 (16)	21 (21)	
No	37 (74)	42 (84)	79 (79)	
Total	50 (100)	50 (100)	100 (100)	
**Gestational age, wk**	10.32 ± 2.45	10.98 ± 2.76		T ^b^ = -1.26 ^a^, df = 98, P = 0.21

^a^ Data are presented as mean ± SD or No. (%)

^b^ Independent sample t-test

**Table 3.** Mean Scores for Nausea and Vomiting Before and Four Days After the Intervention

	Intervention Group (n=50)	Control Group (n = 50)	MD ^a^ (CI95%)	Statistical Indicators
**Before intervention**	8.52 ± 2.27	7/48 ± 1.58	1.04 (0.26, 1.81	T = 2.65 ^b^, df = 87.29, P = 0.01
**First day**	7.44 ± 1.96	7.56 ± 2.27	-0.39 (-1.26, 0.46)	F = 0.84 ^c^, df = 1, P = 0.36
**Second day**	6.70 ± 2.23	7.32 ± 2.23	-1.06 (-1.94, -0.19)	F = 5.85, df = 1, P = 0.017
**Third day**	6.30 ± 2.05	6.76 ± 2.10	-0.74 (-1.56, 0.07)	F = 3.29, df = 1, P = 0.073
**Fourth day**	5.72 ± 2.33)	6.28 ± 2.47	-1.00 ( -1.95, -0.05)	F = 4.39, df = 1, P = 0.039

^a^ Mean Difference (Confidence Interval 95%)

^b^ Independent sample t-test

^c^ ANOVA

**Table 4.** Comparison of Mean Difference of Total Scores of Nausea and Vomiting in Five Intervals ^a^

	Intervention Group	Control Group	MD ^b^ (CI95%)	Statistical Indicators
**1 day after and before intervention**	-1.08 ± 2.40	0.08 ± 2.52	-1.16 (-2.13, -0.18)	T ^c^ = -2.35, df = 97.87, P = 0.02
**2 days after and before intervention**	-1.82 ± 2.47	-0.16 ± ( 2.27	-1.66 (-2.60, -0.71)	T = -3.49, df = 97.30, P = 0.001
**3 days after and before intervention**	-2.22 ± 2.45	-0.72 ± 2.17	-1.50 (-2.42, -0.57)	T = -3.23, df = 96.63, P = 0.002
**4 days after and before intervention**	-2.80 ± 2.7	-1.2 ± 2.32	-1.60 (-2.60, -0.59)	T= -3.17, df = 95.91, P = 0.002
**ANOVA with repeated measures**	F = 6.86, df = 2.96, P < 0.001	F = 2.66, df = 3.50, P = 0.049		

^a^ Data are presented as mean ± SD

^b^ Mean Difference (Confidence Interval 95%)

^c^ Independent sample T-test

## 5. Discussion

According to the findings of the present study, the mean scores of NVP were decreased during four days of using inhalation lemon aromatherapy; however, this reduction in score was statistically significant only in second and forth days of follow up within two groups. Using complementary and alternative medicine (CAM) in Iran has a long history and midwives and physicians are interested in using it. Aromatherapy is a method that in addition to the physical effects, has psychological effects (such as relaxation or stimulation) that can recur very rapidly (16).

Smells in the lowest basic level, can be stimulate the body to respond physically and psychologically. When inhaling aromatic substances such as herbal oil emit odor molecules, the receptor cells in the nasal send impulses directly into olfactory region of the brain. The region is closely related to the other systems that control the memory, emotions, hormones, sex, and heart rate. Impulses act immediately and the released hormones are able to stimulate, appease, calm, or elate the person, leading to the creation of physical and mental changes (10). Lemon aromatherapy can have beneficial effects on NVP (17). Results of a study of Pretest-Posttest in Indonesia, on 12 pregnant women with NVP showed that lemon aromatherapy reduced NVP (18), which is in agreement with the results of this study. Erick et al. investigated women's use of non-pharmacological treatments for the relief of NVP. The results of her study showed that 40% of females used lemon scent to relieve NVP, and 26.5% of those who had used it mentioned it to be effective (11).

Results of a study by Pasha et al. who used peppermint inhaled aromatherapy to relieve NVP on 60 pregnant women showed that mint aromatherapy is not effective in reducing NVP that might be probably due to the small sample size used in their study (19). Results of the study by Mahmoud et al. who used the combination of aromatherapy (essential oils of peppermint and lavender) to relieve NVP suggested that the combination of aromatherapy reduced the severity of NVP, increased energy levels, and reduced fatigue in pregnant women (16). In addition, the study by Lane et al. suggested that inhaled peppermint aromatherapy was effective in reducing nausea and vomiting after cesarean delivery (14). on the other hand, results of the study by Ferruggiari et al. showed no effect of inhaled peppermint aromatherapy on nausea and vomiting after surgery in women (15).

Based on the results of the present study, nausea and vomiting in both groups was reduced by time, but this loss in the intervention group was more significant than in the control group. This decrease in the control group might be due to the placebo effect that is seen in such studies. Placebo effect in studies where subjective outcomes are to be measured is more likely. Because in such studies, the detection of adverse effects arising from reporting bias and the true placebo effect is difficult. As in subjective outcome measures, it is likely that the patient tries to report that the recovery is improved to please the researcher, yet in fact it is not true. the likelihood this type of biases is probably higher in trials that placebo is used only as a treatment (20).

Significant reduction in nausea and vomiting scores only in the second and fourth days between the groups might be due to different response by individuals to the aromatherapy. In other words, this treatment might be pleasant for some and it might be uncomfortable for others. In aromatherapy, patients need to breathe a certain smell based on their psychosocial circumstances, and each individual will react to a certain smell differently (19). Hence, it can be considered as one of the limitations to this study. Another limitation to this study was inability to control the psychological factors during intervention. One of the strengths of this study was the use of PUQE-24 questionnaire that is designed specifically for NVP. We had not found any clinical trials that have been examined the effects of Lemon aromatherapy on NVP. Therefore, due to the limited number of studies in the field of aromatherapy on NVP, there is a need for more research in this field. Inhalation aromatherapy with Lemon essential oil showed that this method could reduce NVP. In contrast to chemical medications, aromatherapy has useful effects on physical and psychological health and might be useful as an alternative approach in the treatment of NVP.
